# Advances in understanding the evolution of fungal genome architecture

**DOI:** 10.12688/f1000research.25424.1

**Published:** 2020-07-27

**Authors:** Shelby J. Priest, Vikas Yadav, Joseph Heitman

**Affiliations:** 1 Department of Molecular Genetics and Microbiology, Duke University Medical Centre, Durham, NC, USA

**Keywords:** Fungal Genetics, Fungal Genomics, Genome Evolution, Genetics, Fungi, Genomics, Evolution

## Abstract

Diversity within the fungal kingdom is evident from the wide range of morphologies fungi display as well as the various ecological roles and industrial purposes they serve. Technological advances, particularly in long-read sequencing, coupled with the increasing efficiency and decreasing costs across sequencing platforms have enabled robust characterization of fungal genomes. These sequencing efforts continue to reveal the rampant diversity in fungi at the genome level. Here, we discuss studies that have furthered our understanding of fungal genetic diversity and genomic evolution. These studies revealed the presence of both small-scale and large-scale genomic changes. In fungi, research has recently focused on many small-scale changes, such as how hypermutation and allelic transmission impact genome evolution as well as how and why a few specific genomic regions are more susceptible to rapid evolution than others. High-throughput sequencing of a diverse set of fungal genomes has also illuminated the frequency, mechanisms, and impacts of large-scale changes, which include chromosome structural variation and changes in chromosome number, such as aneuploidy, polyploidy, and the presence of supernumerary chromosomes. The studies discussed herein have provided great insight into how the architecture of the fungal genome varies within species and across the kingdom and how modern fungi may have evolved from the last common fungal ancestor and might also pave the way for understanding how genomic diversity has evolved in all domains of life.

## Introduction

The fungal kingdom is estimated to consist of 2.2 to 3.8 million different species, making it the most diverse kingdom within the eukaryotic domain ^1^. Fungi are currently organized into eight taxonomically distinct phyla ^2^. The most studied and well-characterized fungi belong largely to the Ascomycota and Basidiomycota phyla, which collectively make up the subkingdom Dikarya. The six most basal phyla, also known as early-diverging fungi, are relatively understudied compared to Dikarya fungi and include the Blastocladiomycota, Chytridiomycota, Mucoromycota, Zoopagomycota, Cryptomycota, and Microsporidia phyla ^2, 3^.

The diversity within the fungal kingdom is evident from genetic, phenotypic, and ecological perspectives. Fungi display a range of morphologies from macroscopic, multicellular filamentous fungi to environmentally ubiquitous, single-celled yeasts and obligate intracellular pathogens associated with animal hosts. This diversity is further demonstrated by the variety of ecological roles fungi fulfill, the importance of fungi as model organisms in scientific research, and the diversity of applications of fungi in industrial settings. The diverse and important roles of fungi combined with technological advances in high-throughput sequencing, also known as next-generation sequencing, have motivated broad efforts to sequence thousands of fungal genomes ^4– 7^. The phenotypic and ecological diversity across the fungal kingdom is reflected, in part, in the diversity observed within fungal genomes.

**Figure 1.**
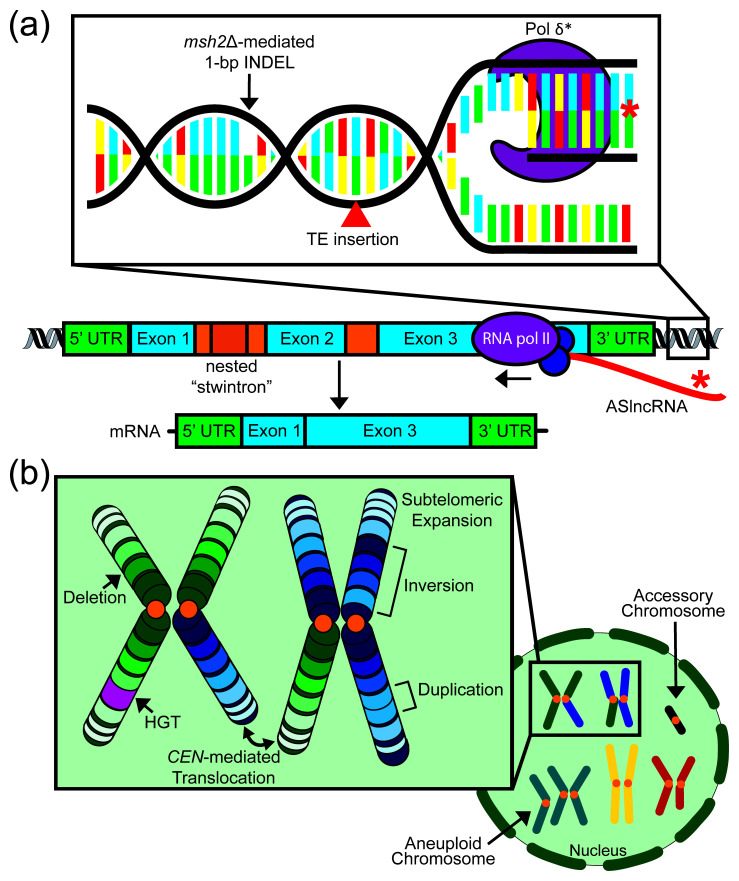
Mechanisms of fungal genome evolution. **(a) Forces driving genomic evolution in fungi at the nucleotide and gene level** Defects in components of the DNA mismatch repair pathway, such as Msh2, contribute to hypermutation. *msh2*Δ mutants display characteristic 1 base pair (bp) insertions or deletions in homopolymeric nucleotide runs. Transposon insertions and the long terminal repeat footprints they leave behind also influence small-scale genomic evolution. Mutations in DNA polymerase delta subunits (Pol δ*) have been observed in fungal hypermutator isolates and typically generate high rates of transition and transversion mutations. At the gene level, nested introns called “stwintrons” have been shown to mobilize within fungal genomes and can trigger alternative splicing and exon skipping. Long non-coding RNAs (lncRNAs), including antisense lncRNAs (ASlncRNAs), undergo rapid evolution and add one more layer of complexity to the genomic evolution of fungi. INDEL, insertion/deletion polymorphism; Pol, polymerase; TE, transposable element; UTR, untranslated region. **(b) Forces driving genomic evolution at the chromosomal and nuclear level** Chromosomal structural changes influencing large-scale genome evolution include deletions, inversions, duplications, and centromere ( *CEN*)-mediated translocations. Interspecies horizontal gene transfer (HGT) and the expansion and contraction of subtelomeric regions also contribute to fungal genome evolution. Karyotypic variations including whole-genome duplication, aneuploidy, and the acquisition of accessory chromosomes represent additional instances of large-scale genomic evolution. Orange circles represent centromeres in both the chromosome and nuclear depictions.

In this review, we focus on recent advances in understanding the evolution of fungal genomes. Both small- and large-scale variation among fungal genomes contributes to stable or transient genotypic and phenotypic variation as well as speciation. Small-scale genomic changes include changes at the nucleotide and gene level, and often influence genomic microevolution, or the changes of allelic frequencies within a species ( Figure 1a). Increased mutation rates and genomic location and context also impact the evolution of fungal genomes ^8– 11^. Introgression, meiotic drive elements, and horizontal gene transfer (HGT) from other fungal species as well as cross-kingdom genetic transfer from prokaryotes represent additional examples of small-scale genomic evolution in fungi ^12– 19^. Compounding elements of small-scale genomic change can mediate genomic macroevolution, which refers to evolution at and above the species level.

Macroevolution of fungal genomes is also influenced by large-scale genomic changes, including structural and organizational changes in individual chromosomes as well as karyotypic variation, which can be attributed to aneuploidy, the acquisition of accessory chromosomes, and polyploidy ^20– 24^ ( Figure 1b).

Advances in sequencing technologies have allowed robust and rapid characterization of these small- and large-scale genomic changes at a more accurate level than previously possible and therefore have provided novel insights into fungal genomic evolution ^25– 28^. These insights contribute to our understanding of fungal genomic evolution and the impacts this evolution can impart on gene function, mating, fitness, and speciation ^29– 31^. Genomic evolutionary mechanisms and dynamics in fungi can provide insight into the evolutionary histories and trajectories of species throughout the eukaryotic domain.

## Small-scale evolution of fungal genomes

Single nucleotide changes, transposition of genetic elements, and expansion and contraction of repetitive sequences influence genome evolution at a small scale. Relatively small-scale genomic changes also include the transfer of genetic information between and among populations. Here we consider advances in understanding how increased mutation rates, fast-evolving genomic regions and elements, and allelic transmission influence the evolution of fungal genomes.

### Hypermutation

Mutation rates estimate the number of genetic changes occurring within a cell per generation. Isolates with significantly higher mutation rates than those of closely related isolates or laboratory reference strains of the same species are said to display a hypermutator phenotype. Increased mutation rates have been extensively characterized in the yeast *Saccharomyces cerevisiae* and have provided the foundation for identifying genetic mechanisms underlying human diseases, including a form of hereditary cancer known as Lynch syndrome ^32– 36^. Hypermutation in fungi has been shown to promote rapid adaptation to novel environmental conditions, to influence overall fitness and genomic structural changes, and to be subsequently tempered by the emergence of anti-mutator suppressor alleles, among other consequences ^8, 9, 37, 38^.

Recently, naturally occurring fungal hypermutator strains have been identified and characterized. Clinically and environmentally isolated strains of the human fungal pathogens *Cryptococcus deuterogattii*, *Cryptococcus neoformans*, and *Candida glabrata* have been found to harbor loss-of-function mutations in DNA mismatch repair components and thus display hypermutator phenotypes ^9, 39– 43^. Defects in the mismatch repair protein Msh2 are especially prevalent and typically produce single base pair insertions or deletions in homopolymeric nucleotide runs ^9, 44^. These isolates display higher rates of genomic evolution at the nucleotide level and have the ability to more rapidly adapt to novel stressors *in vitro*, including the ability to develop *de novo* resistance to antifungal drugs *in vitro* ^9, 39– 42, 45^. Incompatibilities in the mismatch repair components Mlh1 and Pms1 have also been reported to contribute to hypermutation in *S. cerevisiae* laboratory strains ^46, 47^. Studies have identified several *S. cerevisiae* strains isolated from humans that encode these same *MLH1*- *PMS1* incompatible alleles ^48^. Although the *S. cerevisiae* diploids with incompatible alleles isolated from humans were not hypermutators themselves, their meiotic progeny displayed a wide range of mutation rates and included hypermutators, thus providing an opportunity to produce progeny with variable fitness under stressful conditions ^47, 48^. Another mechanism of hypermutation caused by a mutation in a DNA polymerase delta subunit was also described in *C. neoformans*; this mechanism of hypermutation resulted in genome-wide increases in transition and transversion mutations but reduced viability and virulence ^49, 50^.

Mobilization of transposable elements throughout the genome can mediate hypermutation at a larger scale than the single-nucleotide polymorphism changes in mismatch repair and DNA polymerase mutants. Within the past year, two studies have reported instances of hypermutation via mobilization of transposons under stress conditions, illustrating that host infection can trigger transposition in the human pathogen *Cryptococcus deneoformans* and in the plant pathogen *Zymoseptoria tritici* ^51, 52^. Another study has also characterized the evolutionary dynamics and genomic impacts of similar bursts of transposon expansion within the genomes of *Microbotryum* species ^53^. These instances of hypermutation provide important examples of how elevated genome-wide mutation rates influence rapid genomic microevolution, pathogenesis, and the ability of fungi to rapidly adapt to novel environments.

Despite the fitness benefits of hypermutation in stressful or changing conditions, spontaneous mutations can be largely deleterious in normal conditions, and thus hypermutation might be beneficial only from a short-term evolutionary perspective but disadvantageous in the long term ^54^. All organisms, including fungi, must therefore strike a balance between high mutation rates that provide variation for natural selection to act upon and high genomic integrity that allows for long-term survival. Research has shown that, in contrast to hypermutation, some fungal species, particularly those with relatively long lifespans, can have ultra-low mutation rates, such as those observed in the fairy-ring mushroom species *Marasmius oreades* and the pathogenic forest fungus *Armillaria gallica* ^55, 56^. On the other hand, some fungi have been found to persist in the long term with high mutation rates and compromised genome integrity. For example, several novel *Hanseniaspora* species have recently been identified and contributed to the characterization of two *Hanseniaspora* lineages with different rates of evolution: a fast-evolving lineage and a slow-evolving lineage ^57^. While species in both lineages have lost a number of genes involved in regulating the cell cycle and maintaining genomic integrity, the fast-evolving lineage has undergone rapid genomic changes and represents a group of hypermutator species. These species provide a perspective on the long-term evolutionary consequences of sustained elevated mutation rates and could provide answers to critical questions in the field ^57^.

### Fast-evolving regions of the genome

Genomic evolution can occur at different rates that are dependent on genomic context. Rapid evolution within a genome is driven by the adaptive potential of alleles and relaxed selective constraints as well as the propensity of regions to undergo change. Fungal genomic regions that typically experience faster evolution than other loci include introns, intergenic sequence, centromeres, and subtelomeric regions ^58– 65^.

The evolution of introns throughout fungal lineages is dictated by retention of ancestral introns, frequent intron loss events, and relatively few instances of intron gain ^58, 59, 66, 67^. Due to the large variation in intron content across fungal genomes, introns are considered to be one of the more rapidly evolving genomic elements in fungi ^58, 59, 66^. Spliceosomal twin introns or “stwintrons” were recently identified in several orders of ascomycete species ^68, 69^. These nested introns display intriguing trends of gain and loss across fungi and either are removed by complex, multi-step splicing events or trigger alternative splicing and exon skipping. The evolutionary dynamics of these newly identified stwintrons may provide insight into the evolution of intron gain and loss, thus further informing the known evolutionary trajectory of fungal genomes.

The breadth and depth of RNA sequencing in fungi has revealed that long non-coding RNAs (lncRNAs) are abundant and diverse within fungal genomes, are transcribed from intergenic and genic regions alike, and carry out a number of distinct roles ^60, 61, 70^. Only recently have fungal lncRNAs been identified and characterized in fungi other than the model yeasts *S. cerevisiae* and *Schizosaccharomyces pombe*. These newly characterized fungal lncRNA transcriptomes show little conservation between species as well as little consensus in post-transcriptional modifications ^71– 73^. Across fungi, lncRNAs have been shown to regulate gene expression in cis and in trans, influencing mating behaviors, sexual development, morphogenesis, cellular metabolism, virulence, drug resistance, and genomic stability ^61, 70, 74^. One class of lncRNAs are antisense lncRNAs (ASlncRNAs), which overlap with the open-reading frames of genes in an antisense orientation and regulate expression of their cognate transcripts. It has been shown that loss of RNAi in fungi is correlated with expansion and increased abundance of ASlncRNAs, suggesting that these elements play an important role in modulating gene expression and therefore in the stability and evolution of fungal genomes ^75– 77^. Additional deep RNA sequencing among numerous and diverse fungi complemented with molecular genetic analyses will provide further insight into the diversity of lncRNAs and their structure, function, evolution, and impact on overall genomic evolution.

Despite having a conserved role in proper chromosome segregation, centromeres are one of the most rapidly evolving sequences among eukaryotes ^62^. Previous studies focused on centromere identification in relatively few ascomycete fungal species, including *S. cerevisiae*, *S. pombe,* and *Candida albicans* ^78, 79^. These studies revealed that centromere length in fungi could range from a few hundred nucleotides to 300 kb in length and varied significantly among related species. These studies also established that centromeres are defined by the binding of a conserved histone H3 variant, CENP-A, along with many other conserved kinetochore proteins. Over the past several years, whole-genome sequencing with long-read Nanopore and PacBio sequencing platforms has led to the identification of centromeres in many other fungi and a better understanding of centromere structure and evolution. One such study revealed that centromeres across the *Schizosaccharomyces* group harbor a similar architecture but vary in their sequence, suggesting sequence is not critical for centromere function ^25^. Centromeres in the plant-pathogenic ascomycete *Magnaporthe oryzae* were found to consist of long AT-rich sequences, a feature conserved in related species ^26^.

Characterization of centromeres in the *Cryptococcus* pathogenic species complex revealed that centromeres in *Cryptococcus* are long, rich with retrotransposons, and syntenic among different species ^80, 81^. However, centromere lengths vary significantly among three pathogenic species in this complex due to the truncation of retroelements as a result of RNAi loss in one of the species ^81^. A more recent study in another basidiomycete group of *Malassezia* species identified short centromeres (less than 1 kb), which were highly AT-rich and devoid of any repeat content ^82^. Following the pattern in *Schizosaccharomyces* and the *Cryptococcus* species complex, centromeres in *Malassezia* are also syntenic between closely related species.

The nature of centromeres in the Mucoromycota phyla of fungi have also been characterized using *Mucor circinelloides,* an emerging model organism ^83^. It is important to note that *M. circinelloides* lacks CENP-A but harbors other conserved kinetochore proteins. Identification of centromeres in this species revealed a mosaic nature of centromeres where kinetochore binding was restricted to a small region, flanked by multiple copies of a retrotransposon. Each of these studies emphasizes the fact that fungal centromeres are highly diverse with few conserved features and are rapidly evolving, even among closely related species. Interestingly, all centromeres identified thus far are in ORF-free regions that are mostly devoid of transcription, and many centromeres are AT-rich in nature. Combining these features together might help in predicting centromeres in fungal species where experimental tools have not been applied or are not yet available.

Other genomic regions in which rapid evolution has been observed are the subtelomeres and the repeat-rich regions of plant pathogens that harbor effector genes mediating virulence. Subtelomeric loci are prone to rapid expansion and contraction and often encode virulence-associated genes as well as biosynthetic gene clusters for many secondary metabolites. The impact of rapid subtelomeric evolution on virulence has been highlighted in the human fungal pathogens *Candida auris* and *Aspergillus fumigatus* as well as in the plant fungal pathogen *Pyrenophora teres* f. *teres* ^63– 65^. Interestingly, in the emerging fungal pathogen *C. auris*, a particular genetic lineage that has not been involved in outbreaks exhibits high mutation rates and genomic instability, especially in subtelomeric regions. This subtelomeric variation is hypothesized to be attributable to a loss-of-function mutation in *DCC1*, a gene involved in regulating telomere length, genome instability, and the cohesion of sister chromatids in yeast ^64, 84^. Similar to subtelomeric regions, the regions harboring effector genes, also known as effector compartments, in fungal plant pathogens are also prone to frequent expansion and contraction events ^10, 11^. These regions are enriched in transposable elements and are regularly located near chromosome rearrangement breakpoints, which contributes to their largely lineage-specific identity ^11^. Effector compartments make up the rapidly evolving portion of the “two-speed” genomes of many plant fungal pathogens. Rapid evolution in these loci is attributed to the expansion, contraction, and recombination mediated by repetitive transposons within the region as well as repeat-induced mutation (RIP) and epigenetic silencing ^10^.

### Allelic transmission

The transmission of alleles between species can have a profound impact on genomic evolution. Instances of inter- and intra-kingdom HGT, genetic introgression following hybridization, and meiotic drive elements have all been shown to be strong forces of genomic evolution and have thus received increased attention over the last decade. Robust whole-genome sequencing of isolates within a species as well as the increasing diversity of fungal species sequenced have allowed for the identification of these allelic transmission events and mechanisms and also shed light on their origins ^12– 14, 85– 91^.

HGT refers to the lateral exchange and integration of genetic material between two species, unlike the vertical inheritance pattern between parent and offspring ^92^. Previously, prokaryotic species were thought to experience frequent HGT events, while relatively few HGT events were thought to have occurred in fungi. However, advances in whole-genome sequencing have, by increasing efficiency and reducing costs, expanded the number of fungal genome sequences available and allowed the identification of numerous HGT events across the fungal kingdom; many instances of HGT events in fungi have been extensively characterized and reviewed ^16– 19^. HGT events appear to be more frequent in organisms that have undergone significant niche adaptation such as commensal organisms like the saprophytic skin commensal *Malassezia* species, which have acquired a bacterial flavohemoglobin as well as many other bacterially derived genes ^93^. Similarly, obligate intracellular pathogens belonging to the Microsporidia and Cryptomycota phyla have experienced significant HGT, in which up to 2% of genes are estimated to be derived from HGT events ^85^. This work has helped to markedly clarify the number of HGT events in Microsporidia because only a small number of HGT events involving bacteria- and animal-derived genes had been previously documented, and these events were thought to be rare ^16, 94– 96^. In addition to the relatively common HGT of bacterially derived genes in fungi, an exciting new study has identified instances of trans-kingdom genetic transfer between an arbuscular mycorrhizal fungal species and its symbiotic plant partner ^97^. Another case of trans-kingdom HGT was shown in basal fungi (Chytrids) where a key cell-cycle regulator protein evolved from a viral protein ^86^. This study provides key insights into the evolution of cell-cycle regulating machinery that evolved differently in fungi and animals. Further characterization of genomic diversity within and among species across all domains of life will help to identify additional instances of HGT.

Introgression, also known as introgressive hybridization, refers to the integration of genetic alleles from one genetic lineage into another following hybridization. Population-based studies in fungi have revealed the high frequency with which introgression can occur. Two recent examples include 1) the loss and regain of fermentation capacity by *Kluyveromyces lactis,* which was demonstrated to be a result of introgression of a subtelomeric locus from *Kluyveromyces marxianus*, and 2) finding that a lichen-forming *Rhizoplaca* species likely originated from an introgressive hybridization event ^12, 13^. In another intriguing study, introgression following hybridization was shown to be the primary force of evolution in the fungal pathogen responsible for Dutch elm disease, driving changes in host–pathogen interactions via introgression of virulence-associated genes and changes in sexual reproduction via introgression of mating-type loci ^14^.

Population genomics approaches have also revealed the prevalence of meiotic drive elements throughout the fungal kingdom. Meiotic drive elements are genetic elements that cause non-Mendelian patterns of inheritance by either biasing their inheritance in sexual progeny or by killing progeny that do not inherit the element, also known as killer meiotic drive elements. Fungal meiotic drive elements were first identified in *Neurospora* many decades ago ^98^. Subsequent studies have further characterized the meiotic drive elements in *Neurospora*, known as Spore Killers, and have revealed other Spore Killer meiotic drive elements in *Podospora* ^87, 88, 99– 102^. Over the past several years, elegant and thorough research in *Schizosaccharomyces* species has identified several novel meiotic drive elements ^15, 89, 90, 103, 104^. Studies have also shown that in addition to the genomic loci that can act as meiotic drive elements, supernumerary chromosomes in the plant pathogen *Z. tritici* show evidence of meiotic drive ^91^. The ability of genetic elements to bias their inheritance without conferring any adaptive benefits or potentially reducing fitness can greatly influence the genomic landscape and evolutionary trajectory of a species.

## Large-scale evolution of fungal genomes

Instances of large-scale genomic evolution include chromosomal structural rearrangements and numerical increases or decreases in chromosome copy number. Chromosomal structural changes are rearrangements of chromosome segments that result from recombination or DNA damage repair. Numerical alterations include variation in the chromosome number due to aneuploidy or polyploidy as well as changes due to accessory chromosomes. The past several years have seen a significant increase in studies characterizing such events.

### Chromosome structural variations

Structural variations or chromosome rearrangements occur when chromosome segments are shuffled across the genome. These structural variations include deletions, inversions, duplications, and translocations. Chromosome rearrangements are known to occur in all eukaryotic genomes ^105– 107^. These rearrangements can occur within a single chromosome (intra-chromosomal) as well as across different chromosomes (inter-chromosomal). Many different mechanisms are known to mediate rearrangements, including meiotic recombination, double-stranded break (DSB) repair during mitosis, and transposable elements ^108, 109^.

The prevalence and mechanisms of chromosome rearrangements in fungi have been known for a long time, but recent advances in long-read sequencing have highlighted the importance of these events in multiple cellular processes. Several studies comparing the genome evolution of closely related species revealed that chromosome translocations can drive the evolution of secondary metabolite-producing gene clusters, and many of these translocations coincide with transposable elements ^110– 112^. Some of these rearrangements are also correlated with gene loss, the generation of genetic diversity, and changes in pathogenicity ^113^. A study in the basal *Taphrina* genus showed that chromosome rearrangements can drive the evolution of effector superfamily genes and host adaptation as well as speciation ^114^. Another study comparing the genomes of 71 ascomycete species suggests that the rate of chromosome rearrangements accelerated the process of speciation in these species ^115^. A comparison of two different subphyla showed that the number of rearrangements is directly correlated with species richness in each respective subphylum. The same study also showed that whole-genome duplication and pathogenicity might elevate gene order divergence and genome rearrangements ^115^.

Using a direct experimental approach, we recently showed that centromere-mediated chromosome rearrangements, induced by targeting DSBs at centromeric retrotransposons with CRISPR, can alone drive reproductive isolation and thus play a role in speciation ^116^. While centromere-mediated translocation has been proposed to occur based on genome comparisons, we showed its direct impact on the sexual cycle. Such centromere-facilitated chromosome rearrangements have been observed in *Malassezia* and *Candida* and are proposed to play a role in both chromosome and karyotypic evolution in these species ^82, 117, 118^. Centromere-mediated chromosomal translocation has also been proposed as a driving force during the evolution of the mating-type locus in *Cryptococcus* from an ancestral outcrossing tetrapolar system to a derived bipolar inbreeding state ^80^.

Combined, these studies show that structural variations play a critical role in the evolution of a diverse array of functions in fungal species, including metabolism, pathogenicity, host adaptation, and speciation. Their role in the evolution of secondary metabolite-producing clusters has been observed in multiple fungal species complexes, suggesting that this is a common mechanism via which metabolic potential can be altered or expanded. Additionally, the role of chromosome translocation in speciation has received significant support as a result of these studies. Future studies will reveal the unexplored significance of these chromosome structural changes in fungi. One key aspect to focus on will be the impact of these changes on pathogenicity and how they contribute towards the evolution of virulence in fungal pathogens.

### Polyploidy

Whole-genome duplication or polyploidy refers to scenarios in which a cell or organism possesses three or more complete sets of chromosomes compared to the standard haploid karyotype. Polyploidy is found across the fungal kingdom, and one route to its generation involves whole-genome duplication ^23, 24^. Indeed, analysis of the whole-genome sequence of *Saccharomyces* revealed that one such whole-genome duplication event was responsible for the evolution of this genus and likely involved an interspecies hybridization event ^30, 119^. Despite the presence of polyploidy across the fungal kingdom, studies analyzing the polyploidy of fungal genomes have started to re-emerge ^23^. Many clinically relevant fungi show variation in ploidy, mainly in response to stressful or new environmental conditions. Some fungi can also exist in multiple different ploidy states and exhibit ploidy transitions from one state to another. Studies in *S. cerevisiae*, as well as *C. albicans*, found that ploidy variation can drive rapid adaptation in response to environmental conditions and vice versa ^120, 121^.

A well-studied process of ploidy transition is the parasexual cycle in *C. albicans* ^122^. During this process, tetraploid *C. albicans* cells undergo ploidy reduction to generate more stable diploid cells, which can be selectively advantageous ^123^. This process is accompanied by concerted chromosome loss and generates aneuploidy ^124^. Recent studies have provided intriguing insights into this process. Tetraploid *C. albicans* cells were found to be metabolically hyperactive, generating reactive oxygen species and DNA DSBs, resulting in increased genomic instability ^125^. Another study found that the genome reduction in this parasexual cycle involves recombination, similar to meiosis observed in other organisms, and thus has been termed para-meiosis ^126^. A closely related species, *Candida tropicalis,* was found to exhibit a combination of same- and opposite-sex mating to generate polyploidy, which in turn increases genetic diversity ^127^.

Another human fungal pathogen, *C. neoformans*, has been shown to exist in haploid or hybrid diploid states. *C. neoformans* can also exist in a polyploid state, known as titan cells, produced in response to host conditions ^128, 129^. These titan cells produce haploid and aneuploid progeny and thereby enhance stress adaptation ^130^. A concerted effort from three independent studies established that this phenotype can be induced by multiple environmental as well as genetic signals ^131– 133^. Similar to *C. albicans*, the ploidy reduction in these titan cells is mediated by the activation of meiotic genes ^134^. While these studies explore the reduction of a polyploid genome to a more stable state, the exact role of polyploidy and the factors responsible for inducing polyploidy are not well understood. The presence of different levels of ploidy in *S. cerevisiae* natural isolates hints toward its role in adaptation to environmental conditions, but such natural resources have not been explored in other fungal species yet.

### Aneuploidy

Aneuploidy refers to the stage when a cell deviates from its balanced genome by gaining or losing chromosomes. Aneuploidy has been described in a large number of fungi but mainly with respect to stress conditions. While aneuploidy is commonly thought of as a deleterious state, and in humans is invariably associated with disease states or is lethal, many studies have found naturally occurring aneuploid isolates in different fungal species ^20, 24^. It has been proposed that aneuploidy may represent an intermediate, transient state that can promote evolutionary adaptation ^135, 136^. Experimental evolution of multiple *S. cerevisiae* strains showed that aneuploidy frequently occurs during the course of evolution ^137^. Similar results were observed when 47 non-laboratory isolates of *S. cerevisiae* were analyzed ^138^. The gene *SSD1* and its contributions to mitochondrial physiology were identified as the genetic basis of aneuploidy tolerance in these natural isolates ^139^. A genome-wide genetic deletion screen in *S. cerevisiae* found that genes involved in a ubiquitin-mediated proteasomal degradation pathway also confer tolerance to aneuploidy ^140, 141^.

Aneuploidy has been previously known to play a crucial role in drug resistance in pathogenic fungi like *C. albicans* and *C. neoformans* ^142– 144^. In both cases, drug resistance involved amplification of fluconazole target genes like *ERG11* and genes responsible for the expression of drug efflux pumps, either by isochromosome formation in *C. albicans* or aneuploidy in *C. neoformans*. Newer studies have shown that aneuploidy can play roles in osmotic stress, flocculation, and ethanol survival as well as responses to starvation conditions in *S. cerevisiae* ^145– 147^. We have also shown that aneuploidy can be essential to overcome reproductive barriers between two strains harboring chromosomal translocations ^116^. Overall, aneuploidy seems to be a conserved fungal response to stress or harsh environmental conditions. However, the advances discussed here reveal a role for aneuploidy in adaptation to growth conditions as well as pathogenicity in fungal species. With their well-characterized role in drug resistance, mechanisms leading to aneuploidy also present potent targets for drug development.

### B chromosomes

Accessory, supernumerary, or B chromosomes are all different names for extra chromosomes that are present in a cell beyond the reference karyotype. These extra chromosomes segregate in a non-Mendelian fashion, are dispensable, and do not undergo recombination or pairing with the main, essential (also known as A) chromosomes ^21, 22^. Accessory chromosomes are well studied in animals and plants but have also been observed in several plant fungal pathogens. Earlier reports suggested that these chromosomes are required for virulence and can be horizontally transmitted ^148, 149^. More recent studies have focused on the characterization of these chromosomes at the molecular level in *Z. tritici* and *M. oryzae*. In *Z. tritici*, B chromosomes were found to be epigenetically indistinguishable from the core set of chromosomes, whereas the B chromosome (also known as a mini-chromosome) in *M. oryzae* was found to be more enriched with transposons relative to the core chromosomes ^150, 151^. B chromosomes in both of these species were also found to harbor active centromeres ^26, 150^. However, the B chromosomes in *Z. tritici* are unstable and frequently lost during vegetative growth ^152^.

Interestingly, the stability of B chromosomes also depends on the host genotype and thus plays a vital role in host–pathogen interactions ^153^. The mini-chromosome in *M. oryzae* was proposed to be involved in the shuffling of AVR effector genes between the mini-chromosome and core chromosomes, thus aiding in host adaptation ^151^. While the exact function of genes present on these accessory chromosomes is still unknown, they significantly influence the pathogenicity of the fungi in which they are present. For this purpose, an in-depth characterization of accessory chromosomes is essential and should include genetic as well as epigenetic approaches. The identification of B chromosomes in more species will advance the field and may reveal new insights into their roles in pathogenesis, adaptation, evolution, and possibly speciation.

## Role of technological advances in understanding genomic evolution

A multitude of advances in high-throughput sequencing, also known as next-generation sequencing, and molecular biology methods have helped to reveal and characterize the forces driving evolution in fungal genomes. Efficiency and improved affordability of high-throughput whole-genome sequencing on Illumina sequencing platforms have promoted some of the largest advances in our understanding of fungal genome evolution by enabling the sequencing of numerous strains within a single species. The whole-genome data of various isolates within a species provides a pan-genome resource that spans the population variation within a species and also provides an opportunity to assess genomic microevolution and potentially macroevolution from a population genetics and genomics approach. Pan-genome sequencing has been especially helpful for identifying rates and dynamics of intraspecific genomic exchange, including the evolution and propagation of meiotic drive elements, as well as identifying inheritance of interspecific genetic material, including HGT events and instances of hybridization leading to introgression.

The technological advancements of the long-read PacBio and Oxford Nanopore Technologies sequencing platforms have been critical in both further understanding macroevolution in fungi and significantly advancing the identification and characterization of chromosome structural variations. Using these technologies, many fungal genomes were sequenced and compared to develop synteny plots, and these synteny plots have allowed the detection of chromosome translocations. Some of these translocations were found to result in loss or gain of effector genes and also to have contributed to the evolution of secondary metabolite genes. A striking example of the advantages long-read sequencing technologies can have over short-read sequencing techniques was recently demonstrated in *S. pombe*, in which PacBio and Nanopore sequencing platforms were able to accurately predict 16-fold more genomic structural variations than short-read technologies ^27, 28^. Additionally, long sequencing reads often span repetitive regions and transposons, allowing sufficient coverage of unique flanking regions, which is essential for assembling repeat-rich regions of genomes. This particular feature has allowed the identification of centromeres in many fungal species, especially in cases where centromeres harbor repetitive elements or transposons. Another significant feature of long-read sequencing methods is the ability to capture telomere repeat sequences, thus allowing telomere-to-telomere genome assembly. The identification of telomere repeat sequences at the ends of mini-chromosomes or B chromosomes provided support for their existence and helped differentiate these supernumerary elements from core chromosomes. Similarly, it was also helpful in the determination of chromosome number in several species. Another type of technique that has helped to achieve enhanced fungal genome assemblies is chromosome conformation capture, which includes methods such as Hi-C ^154, 155^. By defining the interactions between DNA sequences, Hi-C has aided in resolving genome assembly conflicts, thus improving assemblies ^156, 157^. It is important to note that these long-read sequencing techniques are highly error-prone and thus need to be corrected with less error-prone short-read Illumina sequencing prior to gene prediction or gene calling for annotation.

## Conclusions and future perspectives

Research over the last decade has provided unprecedented insight into the evolution of fungal genomes from both micro- and macro-evolutionary perspectives. The ease, efficiency, and affordability of sequencing combined with great community efforts have promoted research and given rise to findings that illuminate how small-scale genomic changes such as hypermutation, mobilization of various genetic elements, allelic transmission, and rapidly evolving regions can impact the evolution of fungi. Due to advances in long-range whole-genome sequencing technologies and the generation of telomere-to-telomere assemblies, researchers can now characterize the evolution of ploidy and chromosome structure, particularly in highly repetitive regions, with high accuracy and confidence. Advances in understanding fungal genome evolution at the nucleotide, gene, chromosomal, and nuclear levels are illustrated in Figure 1. Long-read sequencing platforms will continue to be important technologies for further characterization of highly repetitive regions, such as centromeres, transposon-rich regions, subtelomeric loci, and telomeric repeats. Studies employing these techniques in a vast array of species will shed light on evolutionarily missing links while also providing a better understanding of already existing processes.

Methods and techniques that attempt to further improve long-range sequencing and its accuracy as well as resolve artifacts and biases generated through library preparation, sequencing itself, and subsequent analyses will also improve our understanding of fungal genome evolution.

Improvements are still especially needed in RNA sequencing and sequencing library preparation. Because of the high abundance of some RNA species, like ribosomal and translational RNAs, it remains difficult to detect rare RNA species, such as non-coding RNAs and alternatively spliced transcripts. Developments on this front will aid in efforts to further identify and characterize lncRNA transcriptomes in fungi and can help to determine how lncRNAs vary at the pan-genome level and influence genomic evolution.

Continued progress with sequencing and other genomic technologies will provide more insight into the driving forces and consequences of genomic evolution. Additional sampling and sequencing of novel fungi and less well-characterized species will provide further insight into genomic diversity in the fungal kingdom. Owing to the variety of fungal niches and lifestyles, sufficiently culturing fungi for sequencing efforts can be tedious and often impossible based on current knowledge and techniques. Therefore, advances in single-cell sequencing methods could be highly advantageous to characterize the genomes of unculturable species or species that are difficult to isolate. This will also help the sequencing and study of fungi beyond Ascomycota and Basidiomycota, which have been the focus of the majority of research until now. Studies involving fungi from Zygomycota and Chytridiomycota are only beginning and are already providing exciting insights into the diversity and uniqueness of these fungal phyla.

In addition to the analyses conducted on the nuclear genomes presented in many of the studies discussed here, it will be interesting to conduct population genomics studies on mitochondrial genomes in parallel to characterize the diversity, inheritance dynamics, and evolution of these important organelles. Research focused on identifying and characterizing fungal-associated viruses, or mycoviruses, is also garnering increased attention, and developments on this subject will be interesting, especially with regard to their propagation dynamics and impacts on host genome evolution. These potential future research avenues present great and exciting opportunities to further advance the field of fungal genetics.

## References

[1] Hawksworth DL Lücking R: Fungal Diversity Revisited: 2.2 to 3.8 Million Species. *Microbiol Spectr.* 2017;5(4). 10.1128/microbiolspec.FUNK-0052-2016 28752818 PMC11687528

[2] Spatafora JW Aime MC Grigoriev IV: The Fungal Tree of Life: From Molecular Systematics to Genome-Scale Phylogenies. *Microbiol Spectr.* 2017;5(5). 10.1128/microbiolspec.FUNK-0053-2016 28917057 PMC11687545

[3] Spatafora JW Chang Y Benny GL: A phylum-level phylogenetic classification of zygomycete fungi based on genome-scale data. *Mycologia.* 2016;108(5):1028–46. 10.3852/16-042 27738200 PMC6078412

[4] Sherman D Durrens P Beyne E: Génolevures: Comparative genomics and molecular evolution of hemiascomycetous yeasts. *Nucleic Acids Res.* 2004;32(Database issue):D315–8. 10.1093/nar/gkh091 14681422 PMC308825

[5] Grigoriev IV Nikitin R Haridas S: MycoCosm portal: Gearing up for 1000 fungal genomes. *Nucleic Acids Res.* 2013;42(Database issue):D699–D704. 10.1093/nar/gkt1183 24297253 PMC3965089

[6] Wu L McCluskey K Desmeth P: The global catalogue of microorganisms 10K type strain sequencing project: Closing the genomic gaps for the validly published prokaryotic and fungi species. *Gigascience.* 2018;7(5): giy026. 10.1093/gigascience/giy026 29718202 PMC5941136

[7] Varga T Krizsán K Földi C: Megaphylogeny resolves global patterns of mushroom evolution. *Nat Ecol Evol.* 2019;3(4):668–78. 10.1038/s41559-019-0834-1 30886374 PMC6443077

[8] Serero A Jubin C Loeillet S: Mutational landscape of yeast mutator strains. *Proc Natl Acad Sci U S A.* 2014;111(5):1897–902. 10.1073/pnas.1314423111 24449905 PMC3918763

[9] Billmyre RB Clancey SA Heitman J: Natural mismatch repair mutations mediate phenotypic diversity and drug resistance in Cryptococcus deuterogattii. *eLife.* 2017;6:e28802. 10.7554/eLife.28802 28948913 PMC5614558

[10] Dong S Raffaele S Kamoun S: The two-speed genomes of filamentous pathogens: Waltz with plants. *Curr Opin Genet Dev.* 2015;35:57–65. 10.1016/j.gde.2015.09.001 26451981

[11] Faino L Seidl MF Shi-Kunne X: Transposons passively and actively contribute to evolution of the two-speed genome of a fungal pathogen. *Genome Res.* 2016;26(8):1091–100. 10.1101/gr.204974.116 27325116 PMC4971763

[12] Varela JA Puricelli M Ortiz-Merino RA: Origin of Lactose Fermentation in Kluyveromyces lactis by Interspecies Transfer of a Neo-functionalized Gene Cluster during Domestication. *Curr Biol.* 2019;29(24):4284–4290.e2. 10.1016/j.cub.2019.10.044 31813610 PMC6926475

[13] Keuler R Garretson A Saunders T: Genome-scale data reveal the role of hybridization in lichen-forming fungi. *Sci Rep.* 2020;10(1):1497. 10.1038/s41598-020-58279-x 32001749 PMC6992703

[14] Hessenauer P Fijarczyk A Martin H: Hybridization and introgression drive genome evolution of Dutch elm disease pathogens. *Nat Ecol Evol.* 2020;4(4):626–38. 10.1038/s41559-020-1133-6 32123324

[15] Bravo Núñez MA Nuckolls NL Zanders SE: Genetic Villains: Killer Meiotic Drivers. *Trends Genet.* 2018;34(6):424–33. 10.1016/j.tig.2018.02.003 29499907 PMC5959745

[16] Marcet-Houben M Gabaldón T: Acquisition of prokaryotic genes by fungal genomes. *Trends Genet.* 2010;26(1):5–8. 10.1016/j.tig.2009.11.007 19969385

[17] Richards TA: Genome Evolution: Horizontal Movements in the Fungi. *Curr Biol.* 2011;21(4):R166–R168. 10.1016/j.cub.2011.01.028 21334300

[18] Richards TA Leonard G Soanes DM: Gene transfer into the fungi. *Fungal Biol Rev.* 2011;25(2):98–110. 10.1016/j.fbr.2011.04.003

[19] Fitzpatrick DA: Horizontal gene transfer in fungi. *FEMS Microbiol Lett.* 2012;329(1):1–8. 10.1111/j.1574-6968.2011.02465.x 22112233

[20] Tsai HJ Nelliat A: A Double-Edged Sword: Aneuploidy is a Prevalent Strategy in Fungal Adaptation. *Genes (Basel).* 2019;10(10):787. 10.3390/genes10100787 31658789 PMC6826469

[21] Bertazzoni S Williams AH Jones DA: Accessories Make the Outfit: Accessory Chromosomes and Other Dispensable DNA Regions in Plant-Pathogenic Fungi. *Mol Plant Microbe Interact.* 2018;31(8):779–88. 10.1094/MPMI-06-17-0135-FI 29664319

[22] Ahmad S Martins C: The Modern View of B Chromosomes Under the Impact of High Scale Omics Analyses. *Cells.* 2019;8(2):156. 10.3390/cells8020156 30781835 PMC6406668

[23] Albertin W Marullo P: Polyploidy in fungi: Evolution after whole-genome duplication. *Proc Biol Sci.* 2012;279(1738):2497–509. 10.1098/rspb.2012.0434 22492065 PMC3350714

[24] Todd RT Forche A Selmecki A: Ploidy Variation in Fungi: Polyploidy, Aneuploidy, and Genome Evolution. *Microbiol Spectr.* 2017;5(4). 10.1128/microbiolspec.FUNK-0051-2016 28752816 PMC5656283

[25] Tong P Pidoux AL Toda NRT: Interspecies conservation of organisation and function between nonhomologous regional centromeres. *Nat Commun.* 2019;10(1):2343. 10.1038/s41467-019-09824-4 31138803 PMC6538654

[26] Yadav V Yang F Reza MH: Cellular Dynamics and Genomic Identity of Centromeres in Cereal Blast Fungus. *mBio.* 2019;10(4):e01581–19. 10.1128/mBio.01581-19 31363034 PMC6667624

[27] Jeffares DC Jolly C Hoti M: Transient structural variations have strong effects on quantitative traits and reproductive isolation in fission yeast. *Nat Commun.* 2017;8:14061. 10.1038/ncomms14061 28117401 PMC5286201

[28] Tusso S Nieuwenhuis BPS Sedlazeck FJ: Ancestral Admixture Is the Main Determinant of Global Biodiversity in Fission Yeast. *Mol Biol Evol.* 2019;36(9):1975–89. 10.1093/molbev/msz126 31225876 PMC6736153

[29] Stajich JE: Fungal Genomes and Insights into the Evolution of the Kingdom. *Microbiol Spectr.* 2017;5(4). 10.1128/microbiolspec.FUNK-0055-2016 28820125 PMC6078396

[30] Marcet-Houben M Gabaldón T: Beyond the Whole-Genome Duplication: Phylogenetic Evidence for an Ancient Interspecies Hybridization in the Baker’s Yeast Lineage. *PLoS Biol.* 2015;13(8):e1002220. 10.1371/journal.pbio.1002220 26252497 PMC4529251

[31] Sun S Coelho MA Heitman J: Convergent evolution of linked mating-type loci in basidiomycete fungi. *PLoS Genet.* 2019;15(9):e1008365. 10.1371/journal.pgen.1008365 31490920 PMC6730849

[32] Hawk JD Stefanovic L Boyer JC: Variation in efficiency of DNA mismatch repair at different sites in the yeast genome. *Proc Natl Acad Sci U S A.* 2005;102(24):8639–43. 10.1073/pnas.0503415102 15932942 PMC1150857

[33] Lang GI Parsons L Gammie AE: Mutation rates, spectra, and genome-wide distribution of spontaneous mutations in mismatch repair deficient yeast. *G3 (Bethesda).* 2013;3(9):1453–65. 10.1534/g3.113.006429 23821616 PMC3755907

[34] Gou L Bloom JS Kruglyak L: The Genetic Basis of Mutation Rate Variation in Yeast. *Genetics.* 2019;211(2):731–740. 10.1534/genetics.118.301609 30504363 PMC6366923

[35] Strand M Prolla TA Liskay RM: Destabilization of tracts of simple repetitive DNA in yeast by mutations affecting DNA mismatch repair. *Nature.* 1993;365(6443):274–6. 10.1038/365274a0 8371783

[36] Aldred PMR Borts RH: Humanizing mismatch repair in yeast: Towards effective identification of hereditary non-polyposis colorectal cancer alleles. *Biochem Soc Trans.* 2007;35(Pt 6):1525–8. 10.1042/BST0351525 18031259

[37] Thompson DA Desai MM Murray AW: Ploidy controls the success of mutators and nature of mutations during budding yeast evolution. *Curr Biol.* 2006;16(16):1581–90. 10.1016/j.cub.2006.06.070 16920619

[38] McDonald MJ Hsieh YY Yu YH: The Evolution of Low Mutation Rates in Experimental Mutator Populations of *Saccharomyces cerevisiae*. *Curr Biol.* 2012;22(13):1235–40. 10.1016/j.cub.2012.04.056 22727704

[39] Billmyre RB Croll D Li W: Highly recombinant VGII Cryptococcus gattii population develops clonal outbreak clusters through both sexual macroevolution and asexual microevolution. *mBio.* 2014;5(4):e01494–14. 10.1128/mBio.01494-14 25073643 PMC4128362

[40] Boyce KJ Wang Y Verma S: Mismatch Repair of DNA Replication Errors Contributes to Microevolution in the Pathogenic Fungus *Cryptococcus neoformans*. *mBio.* 2017;8(3):e00595–17. 10.1128/mBio.00595-17 28559486 PMC5449657

[41] Rhodes J Beale MA Vanhove M: A Population Genomics Approach to Assessing the Genetic Basis of Within-Host Microevolution Underlying Recurrent Cryptococcal Meningitis Infection. *G3 (Bethesda).* 2017;7(4):1165–1176. 10.1534/g3.116.037499 28188180 PMC5386865

[42] Healey KR Zhao Y Perez WB: Prevalent mutator genotype identified in fungal pathogen *Candida glabrata* promotes multi-drug resistance. *Nat Commun.* 2016;7:11128. 10.1038/ncomms11128 27020939 PMC5603725

[43] Singh A Healey KR Yadav P: Absence of Azole or Echinocandin Resistance in *Candida glabrata* Isolates in India despite Background Prevalence of Strains with Defects in the DNA Mismatch Repair Pathway. *Antimicrob Agents Chemother.* 2018;62(6):e00195–18. 10.1128/AAC.00195-18 29610199 PMC5971596

[44] Tran HT Keen JD Kricker M: Hypermutability of homonucleotide runs in mismatch repair and DNA polymerase proofreading yeast mutants. *Mol Cell Biol.* 1997;17(5):2859–65. 10.1128/mcb.17.5.2859 9111358 PMC232138

[45] Billmyre RB Applen Clancey S Li LX: 5-fluorocytosine resistance is associated with hypermutation and alterations in capsule biosynthesis in *Cryptococcus*. *Nat Commun.* 2020;11(1):127. 10.1038/s41467-019-13890-z 31913284 PMC6949227

[46] Heck JA Argueso JL Gemici Z: Negative epistasis between natural variants of the *Saccharomyces cerevisiae MLH1* and *PMS1* genes results in a defect in mismatch repair. *Proc Natl Acad Sci U S A.* 2006;103(9):3256–61. 10.1073/pnas.0510998103 16492773 PMC1413905

[47] Bui DT Dine E Anderson JB: A Genetic Incompatibility Accelerates Adaptation in Yeast. *PLoS Genet.* 2015;11(7):e1005407. 10.1371/journal.pgen.1005407 26230253 PMC4521705

[48] Raghavan V Bui DT Al-Sweel N: Incompatibilities in Mismatch Repair Genes *MLH1-PMS1* Contribute to a Wide Range of Mutation Rates in Human Isolates of Baker’s Yeast. *Genetics.* 2018;210(4):1253–1266. 10.1534/genetics.118.301550 30348651 PMC6283166

[49] Magditch DA Liu TB Xue C: DNA mutations mediate microevolution between host-adapted forms of the pathogenic fungus *Cryptococcus neoformans*. *PLoS Pathog.* 2012;8(10):e1002936. 10.1371/journal.ppat.1002936 23055925 PMC3464208

[50] Boyce KJ Cao C Xue C: A spontaneous mutation in DNA polymerase *POL3* during *in vitro* passaging causes a hypermutator phenotype in *Cryptococcus* species. *DNA Repair (Amst).* 2020;86:102751. 10.1016/j.dnarep.2019.102751 31838381 PMC7542539

[51] Gusa A Williams JD Cho J-E: Transposon mobilization in the human fungal pathogen *Cryptococcus* is mutagenic during infection and promotes drug resistance *in vitro*. *Proc Natl Acad Sci U S A.* 2020;117(18):9973–9980. 10.1073/pnas.2001451117 32303657 PMC7211991

[52] Fouché S Badet T Oggenfuss U: Stress-Driven Transposable Element De-repression Dynamics and Virulence Evolution in a Fungal Pathogen. *Mol Biol Evol.* 2020;37(1):221–239. 10.1093/molbev/msz216 31553475

[53] Horns F Petit E Hood ME: Massive Expansion of Gypsy-Like Retrotransposons in Microbotryum Fungi. *Genome Biol Evol.* 2017;9(2):363–371. 10.1093/gbe/evx011 28164239 PMC5381629

[54] Eyre-Walker A Keightley PD: The distribution of fitness effects of new mutations. *Nat Rev Genet.* 2007;8(8):610–8. 10.1038/nrg2146 17637733

[55] Hiltunen M Grudzinska-Sterno M Wallerman O: Maintenance of High Genome Integrity over Vegetative Growth in the Fairy-Ring Mushroom Marasmius oreades. *Curr Biol.* 2019;29(16):2758–2765.e6. 10.1016/j.cub.2019.07.025 31402298

[56] Anderson JB Bruhn JN Kasimer D: Clonal evolution and genome stability in a 2500-year-old fungal individual. *Proc Biol Sci.* 2018;285(1893):20182233. 10.1098/rspb.2018.2233 30963893 PMC6304041

[57] Steenwyk JL Opulente DA Kominek J: Extensive loss of cell-cycle and DNA repair genes in an ancient lineage of bipolar budding yeasts. *PLoS Biol.* 2019;17(5):e3000255. 10.1371/journal.pbio.3000255 31112549 PMC6528967

[58] Stajich JE Dietrich FS Roy SW: Comparative genomic analysis of fungal genomes reveals intron-rich ancestors. *Genome Biol.* 2007;8(10):R223. 10.1186/gb-2007-8-10-r223 17949488 PMC2246297

[59] Croll D McDonald BA: Intron gains and losses in the evolution of Fusarium and Cryptococcus fungi. *Genome Biol Evol.* 2012;4(11):1148–61. 10.1093/gbe/evs091 23054310 PMC3514964

[60] Ponting CP Oliver PL Reik W: Evolution and Functions of Long Noncoding RNAs. *Cell.* 2009;136(4):629–41. 10.1016/j.cell.2009.02.006 19239885

[61] Till P Mach RL Mach-Aigner AR: A current view on long noncoding RNAs in yeast and filamentous fungi. *Appl Microbiol Biotechnol.* 2018;102(17):7319–31. 10.1007/s00253-018-9187-y 29974182 PMC6097775

[62] Henikoff S Ahmad K Malik HS: The centromere paradox: Stable inheritance with rapidly evolving DNA. *Science.* 2001;293(5532):1098–102. 10.1126/science.1062939 11498581

[63] Kowalski CH Kerkaert JD Liu KW: Fungal biofilm morphology impacts hypoxia fitness and disease progression. *Nat Microbiol.* 2019;4(12):2430–41. 10.1038/s41564-019-0558-7 31548684 PMC7396965

[64] Munoz JF Welsh RM Shea T: Chromosomal rearrangements and loss of subtelomeric adhesins linked to clade-specific phenotypes in Candida auris. *bioRxiv.* 2020 10.1101/754143 PMC8128392 33769478

[65] Wyatt NA Richards JK Brueggeman RS: A Comparative Genomic Analysis of the Barley Pathogen *Pyrenophora teres* f. *teres* Identifies Subtelomeric Regions as Drivers of Virulence. *Mol Plant Microbe Interact.* 2020;33(2):173–88. 10.1094/MPMI-05-19-0128-R 31502507

[66] Nielsen CB Friedman B Birren B: Patterns of Intron Gain and Loss in Fungi. *PLoS Biol.* 2004;2(12):e422. 10.1371/journal.pbio.0020422 15562318 PMC532390

[67] Sun Y Whittle CA Corcoran P: Intron evolution in Neurospora: The role of mutational bias and selection. *Genome Res.* 2015;25(1):100–10. 10.1101/gr.175653.114 25342722 PMC4317165

[68] Flipphi M Ág N Karaffa L: Emergence and loss of spliceosomal twin introns. *Fungal Biol Biotechnol.* 2017;4:7. 10.1186/s40694-017-0037-y 29046814 PMC5639578

[69] Kavalecz N Ág N Karaffa L: A spliceosomal twin intron (stwintron) participates in both exon skipping and evolutionary exon loss. *Sci Rep.* 2019;9(1):9940. 10.1038/s41598-019-46435-x 31289343 PMC6616335

[70] Niederer RO Hass EP Zappulla DC: Long Noncoding RNAs in the Yeast *S. cerevisiae*. *Adv Exp Med Biol.* 2017;1008:119–32. 10.1007/978-981-10-5203-3_4 28815538

[71] Till P Pucher ME Mach RL: A long noncoding RNA promotes cellulase expression in *Trichoderma reesei*. *Biotechnol Biofuels.* 2018;11:78. 10.1186/s13068-018-1081-4 29588663 PMC5865335

[72] Kim W Miguel-Rojas C Wang J: Developmental Dynamics of Long Noncoding RNA Expression during Sexual Fruiting Body Formation in *Fusarium graminearum*. *mBio.* 2018;9(4):e01292–18. 10.1128/mBio.01292-18 30108170 PMC6094484

[73] Borgognone A Sanseverino W Cigliano RA: Distribution, Characteristics, and Regulatory Potential of Long Noncoding RNAs in Brown-Rot Fungi. *Int J Genomics.* 2019;2019:9702342. 10.1155/2019/9702342 31192251 PMC6525899

[74] Chang Z Yadav V Lee SC: Epigenetic mechanisms of drug resistance in fungi. *Fungal Genet Biol.* 2019;132:103253. 10.1016/j.fgb.2019.103253 31325489 PMC6858951

[75] Alcid EA Tsukiyama T: Expansion of antisense lncRNA transcriptomes in budding yeast species since the loss of RNAi. *Nat Struct Mol Biol.* 2016;23(5):450–5. 10.1038/nsmb.3192 27018804 PMC4856548

[76] Drummond DA Wilke CO: Mistranslation-induced protein misfolding as a dominant constraint on coding-sequence evolution. *Cell.* 2008;134:341–52. 10.1016/j.cell.2008.05.042 18662548 PMC2696314

[77] Drummond DA Wilke CO: The evolutionary consequences of erroneous protein synthesis. *Nat Rev Genet.* 2009;10(10):715–24. 10.1038/nrg2662 19763154 PMC2764353

[78] Roy B Sanyal K: Diversity in requirement of genetic and epigenetic factors for centromere function in fungi. *Eukaryotic Cell.* 2011;10:1384–95. 10.1128/EC.05165-11 21908596 PMC3209047

[79] Yadav V Sreekumar L Guin K: Five pillars of centromeric chromatin in fungal pathogens. *PLoS Pathog.* 2018;14(8):e1007150. 10.1371/journal.ppat.1007150 30138484 PMC6107279

[80] Sun S Yadav V Billmyre RB: Fungal genome and mating system transitions facilitated by chromosomal translocations involving intercentromeric recombination. *PLoS Biol.* 2017;15(8):e2002527. 10.1371/journal.pbio.2002527 28800596 PMC5568439

[81] Yadav V Sun S Billmyre RB: RNAi is a critical determinant of centromere evolution in closely related fungi. *Proc Natl Acad Sci U S A.* 2018;115(12):3108–13. 10.1073/pnas.1713725115 29507212 PMC5866544

[82] Sankaranarayanan SR Ianiri G Coelho MA: Loss of centromere function drives karyotype evolution in closely related *Malassezia* species. *eLife.* 2020;9:e53944. 10.7554/eLife.53944 31958060 PMC7025860

[83] Navarro-Mendoza MI Pérez-Arques C Panchal S: Early Diverging Fungus *Mucor circinelloides* Lacks Centromeric Histone CENP-A and Displays a Mosaic of Point and Regional Centromeres. *Curr Biol.* 2019;29(22):3791–3802.e6. 10.1016/j.cub.2019.09.024 31679929 PMC6925572

[84] Yuen KWY Warren CD Chen O: Systematic genome instability screens in yeast and their potential relevance to cancer. *Proc Natl Acad Sci U S A.* 2007;104(10):3925–30. 10.1073/pnas.0610642104 17360454 PMC1820685

[85] Alexander WG Wisecaver JH Rokas A: Horizontally acquired genes in early-diverging pathogenic fungi enable the use of host nucleosides and nucleotides. *Proc Natl Acad Sci U S A.* 2016;113(15):4116–21. 10.1073/pnas.1517242113 27035945 PMC4839431

[86] Medina EM Turner JJ Gordân R: Punctuated evolution and transitional hybrid network in an ancestral cell cycle of fungi. *eLife.* 2016;5:e09492. 10.7554/eLife.09492 27162172 PMC4862756

[87] Svedberg J Hosseini S Chen J: Convergent evolution of complex genomic rearrangements in two fungal meiotic drive elements. *Nat Commun.* 2018;9(1):4242. 10.1038/s41467-018-06562-x 30315196 PMC6185902

[88] Vogan AA Ament-Velásquez SL Granger-Farbos A: Combinations of *Spok* genes create multiple meiotic drivers in *Podospora*. *eLife.* 2019;8:e46454. 10.7554/eLife.46454 31347500 PMC6660238

[89] Hu W Jiang ZD Suo F: A large gene family in fission yeast encodes spore killers that subvert Mendel's law. *eLife.* 2017;6:e26057. 10.7554/eLife.26057 28631610 PMC5478263

[90] Eickbush MT Young JM Zanders SE: Killer Meiotic Drive and Dynamic Evolution of the wtf Gene Family. *Mol Biol Evol.* 2019;36(6):1201–14. 10.1093/molbev/msz052 30991417 PMC6526906

[91] Habig M Kema GH Holtgrewe Stukenbrock E: Meiotic drive of female-inherited supernumerary chromosomes in a pathogenic fungus. *eLife.* 2018;7:e40251. 10.7554/eLife.40251 30543518 PMC6331196

[92] Syvanen M: Cross-species gene transfer; implications for a new theory of evolution. *J Theor Biol.* 1985;112(2):333–43. 10.1016/s0022-5193(85)80291-5 2984477

[93] Ianiri G Coelho MA Fuchti F: HGT in the human and skin commensal *Malassezia*: a bacterially-derived flavohemoglobin is required for NO resistance and host interaction.(in press at Proc Natl Acad Sci U S A), *bioRxiv.*2020.01.28.923367. 10.1101/2020.01.28.923367 PMC7354939 32576698

[94] Selman M Pombert JF Solter L: Acquisition of an animal gene by microsporidian intracellular parasites. *Curr Biol.* 2011;21(15):R576–7. 10.1016/j.cub.2011.06.017 21820617 PMC3751409

[95] Pombert JF Selman M Burki F: Gain and loss of multiple functionally related, horizontally transferred genes in the reduced genomes of two microsporidian parasites. *Proc Natl Acad Sci U S A.* 2012;109(31):12638–43. 10.1073/pnas.1205020109 22802648 PMC3412028

[96] Nakjang S Williams TA Heinz E: Reduction and expansion in microsporidian genome evolution: New insights from comparative genomics. *Genome Biol Evol.* 2013;5(12):2285–303. 10.1093/gbe/evt184 24259309 PMC3879972

[97] Li M Zhao J Tang N: Horizontal Gene Transfer From Bacteria and Plants to the Arbuscular Mycorrhizal Fungus *Rhizophagus irregularis*. *Front Plant Sci.* 2018;9:701. 10.3389/fpls.2018.00701 29887874 PMC5982333

[98] Turner BC Perkins DD: Spore killer, a chromosomal factor in neurospora that kills meiotic products not containing it. *Genetics.* 1979;93(3):587–606. 17248973 10.1093/genetics/93.3.587 PMC1214100

[99] Hammond TM Rehard DG Xiao H: Molecular dissection of *Neurospora* Spore killer meiotic drive elements. *Proc Natl Acad Sci U S A.* 2012;109(30):12093–8. 10.1073/pnas.1203267109 22753473 PMC3409728

[100] Harvey AM Rehard DG Groskreutz KM: A critical component of meiotic drive in *Neurospora* is located near a chromosome rearrangement. *Genetics.* 2014;197(4):1165–74. 10.1534/genetics.114.167007 24931406 PMC4125391

[101] Rhoades NA Harvey AM Samarajeewa DA: Identification of *rfk-1*, a Meiotic Driver Undergoing RNA Editing in *Neurospora*. *Genetics.* 2019;212(1):93–110. 10.1534/genetics.119.302122 30918007 PMC6499513

[102] Grognet P Lalucque H Malagnac F: Genes that bias Mendelian segregation. *PLoS Genet.* 2014;10(5):e1004387. 10.1371/journal.pgen.1004387 24830502 PMC4022471

[103] Zanders SE Eickbush MT Yu JS: Genome rearrangements and pervasive meiotic drive cause hybrid infertility in fission yeast. *eLife.* 2014;3:e02630. 10.7554/eLife.02630 24963140 PMC4066438

[104] Nuckolls NL Bravo Núñez MA Eickbush MT: *wtf* genes are prolific dual poison-antidote meiotic drivers. *eLife.* 2017;6:e26033. 10.7554/eLife.26033 28631612 PMC5478261

[105] Le Scouarnec S Gribble SM: Characterising chromosome rearrangements: recent technical advances in molecular cytogenetics. *Heredity (Edinb).* 2012;108(1):75–85. 10.1038/hdy.2011.100 22086080 PMC3238113

[106] Weckselblatt B Rudd MK: Human Structural Variation: Mechanisms of Chromosome Rearrangements. *Trends Genet.* 2015;31(10):587–99. 10.1016/j.tig.2015.05.010 26209074 PMC4600437

[107] Zhang Y: Chromosome Translocation. 1st ed. Singapore: Springer;2018 Reference Source

[108] Stukenbrock EH Croll D: The evolving fungal genome. *Fungal Biology Reviews.* 2014;28(1):1–12. 10.1016/j.fbr.2014.02.001

[109] Mehrabi R Mirzadi Gohari A Kema GHJ: Karyotype Variability in Plant-Pathogenic Fungi. *Annu Rev Phytopathol.* 2017;55:483–503. 10.1146/annurev-phyto-080615-095928 28777924

[110] Moolhuijzen P See PT Hane JK: Comparative genomics of the wheat fungal pathogen Pyrenophora tritici-repentis reveals chromosomal variations and genome plasticity. *BMC Genomics.* 2018;19(1):279. 10.1186/s12864-018-4680-3 29685100 PMC5913888

[111] Olarte RA Menke J Zhang Y: Chromosome rearrangements shape the diversification of secondary metabolism in the cyclosporin producing fungus *Tolypocladium inflatum*. *BMC Genomics.* 2019;20(1):120. 10.1186/s12864-018-5399-x 30732559 PMC6367777

[112] Tralamazza SM Rocha LO Oggenfuss U: Complex evolutionary origins of specialized metabolite gene cluster diversity among the plant pathogenic fungi of the Fusarium graminearum species complex. *Genome Biol Evol.* 2019 10.1101/639641 PMC6836718 31609418

[113] Shi-Kunne X Faino L van den Berg GCM: Evolution within the fungal genus *Verticillium* is characterized by chromosomal rearrangement and gene loss. *Environ Microbiol.* 2018;20(4):1362–1373. 10.1111/1462-2920.14037 29282842

[114] Wang Q Sun M Zhang Y: Extensive chromosomal rearrangements and rapid evolution of novel effector superfamilies contribute to host adaptation and speciation in the basal ascomycetous fungi. *Mol Plant Pathol.* 2020;21(3):330–348. 10.1111/mpp.12899 31916390 PMC7036362

[115] Rajeh A Lv J Lin Z: Heterogeneous rates of genome rearrangement contributed to the disparity of species richness in Ascomycota. *BMC Genomics.* 2018;19:282 10.1186/s12864-018-4683-0 29690866 PMC5937819

[116] Yadav V Sun S Coelho MA: Centromere scission drives chromosome shuffling and reproductive isolation. *Proc Natl Acad Sci U S A.* 2020;117(14):7917–7928. 10.1073/pnas.1918659117 32193338 PMC7149388

[117] Chatterjee G Sankaranarayanan SR Guin K: Repeat-Associated Fission Yeast-Like Regional Centromeres in the Ascomycetous Budding Yeast *Candida tropicalis*. *PLoS Genet.* 2016;12(2):e1005839. 10.1371/journal.pgen.1005839 26845548 PMC4741521

[118] Guin K Chen Y Mishra R: Spatial inter-centromeric interactions facilitated the emergence of evolutionary new centromeres. *eLife.* 2020;9:e58556. 10.7554/eLife.58556 32469306 PMC7292649

[119] Wolfe KH Shields DC: Molecular evidence for an ancient duplication of the entire yeast genome. *Nature.* 1997;387(6634):708–13. 10.1038/42711 9192896

[120] Selmecki AM Maruvka YE Richmond PA: Polyploidy can drive rapid adaptation in yeast. *Nature.* 2015;519:349–52. 10.1038/nature14187 25731168 PMC4497379

[121] Gerstein AC Lim H Berman J: Ploidy tug-of-war: Evolutionary and genetic environments influence the rate of ploidy drive in a human fungal pathogen. *Evolution.* 2017;71(4):1025–38. 10.1111/evo.13205 28195309 PMC7035954

[122] Bennett RJ: The parasexual lifestyle of Candida albicans. *Curr Opin Microbiol.* 2015;28:10–7. 10.1016/j.mib.2015.06.017 26210747 PMC4688137

[123] Zhang N Magee BB Magee PT: Selective Advantages of a Parasexual Cycle for the Yeast Candida albicans. *Genetics.* 2015;200(4):1117–32. 10.1534/genetics.115.177170 26063661 PMC4574235

[124] Hickman MA Paulson C Dudley A: Parasexual Ploidy Reduction Drives Population Heterogeneity Through Random and Transient Aneuploidy in Candida albicans. *Genetics.* 2015;200(3):781–94. 10.1534/genetics.115.178020 25991822 PMC4512543

[125] Thomson GJ Hernon C Austriaco N: Metabolism-induced oxidative stress and DNA damage selectively trigger genome instability in polyploid fungal cells. *EMBO J.* 2019;38(19):e101597. 10.15252/embj.2019101597 31448850 PMC6769381

[126] Anderson MZ Thomson GJ Hirakawa MP: A 'parameiosis' drives depolyploidization and homologous recombination in Candida albicans. *Nat Commun.* 2019;10(1):4388. 10.1038/s41467-019-12376-2 31558727 PMC6763455

[127] Du H Zheng Q Bing J: A coupled process of same- and opposite-sex mating generates polyploidy and genetic diversity in Candida tropicalis. *PLoS Genet.* 2018;14(5):e1007377. 10.1371/journal.pgen.1007377 29734333 PMC5957450

[128] Okagaki LH Strain AK Nielsen JN: Cryptococcal cell morphology affects host cell interactions and pathogenicity. *PLoS Pathog.* 2010;6(6):e1000953. 10.1371/journal.ppat.1000953 20585559 PMC2887476

[129] Zaragoza O García-Rodas R Nosanchuk JD: Fungal cell gigantism during mammalian infection. *PLoS Pathog.* 2010;6(6):e1000945. 10.1371/journal.ppat.1000945 20585557 PMC2887474

[130] Gerstein AC Fu MS Mukaremera L: Polyploid Titan Cells Produce Haploid and Aneuploid Progeny To Promote Stress Adaptation. *mBio.* 2015;6(5):e01340–15. 10.1128/mBio.01340-15 26463162 PMC4620463

[131] Dambuza IM Drake T Chapuis A: The *Cryptococcus neoformans* Titan cell is an inducible and regulated morphotype underlying pathogenesis. *PLoS Pathog.* 2018;14(5):e1006978. 10.1371/journal.ppat.1006978 29775474 PMC5959070

[132] Hommel B Mukaremera L Cordero RJB: Titan cells formation in *Cryptococcus neoformans* is finely tuned by environmental conditions and modulated by positive and negative genetic regulators. *PLoS Pathog.* 2018;14(5):e1006982. 10.1371/journal.ppat.1006982 29775480 PMC5959062

[133] Trevijano-Contador N de Oliveira HC García-Rodas R: *Cryptococcus neoformans* can form titan-like cells *in vitro* in response to multiple signals. *PLoS Pathog.* 2018;14(5):e1007007. 10.1371/journal.ppat.1007007 29775477 PMC5959073

[134] Zhao Y Wang Y Upadhyay S: Activation of Meiotic Genes Mediates Ploidy Reduction during Cryptococcal Infection. *Curr Biol.* 2020;30(8):1387–1396.e5. 10.1016/j.cub.2020.01.081 32109388 PMC7228024

[135] Rancati G Pavelka N Fleharty B: Aneuploidy underlies rapid adaptive evolution of yeast cells deprived of a conserved cytokinesis motor. *Cell.* 2008;135(5):879–93. 10.1016/j.cell.2008.09.039 19041751 PMC2776776

[136] Yona AH Manor YS Herbst RH: Chromosomal duplication is a transient evolutionary solution to stress. *Proc Natl Acad Sci U S A.* 2012;109(51):21010–5. 10.1073/pnas.1211150109 23197825 PMC3529009

[137] Zhu YO Siegal ML Hall DW: Precise estimates of mutation rate and spectrum in yeast. *Proc Natl Acad Sci U S A.* 2014;111(22):E2310–8. 10.1073/pnas.1323011111 24847077 PMC4050626

[138] Hose J Yong CM Sardi M: Dosage compensation can buffer copy-number variation in wild yeast. *eLife.* 2015;4:e05462. 10.7554/eLife.05462 25955966 PMC4448642

[139] Hose J Escalante LE Clowers KJ: The genetic basis of aneuploidy tolerance in wild yeast. *eLife.* 2020;9:e52063. 10.7554/eLife.52063 31909711 PMC6970514

[140] Torres EM Dephoure N Panneerselvam A: Identification of aneuploidy-tolerating mutations. *Cell.* 2010;143(1):71–83. 10.1016/j.cell.2010.08.038 20850176 PMC2993244

[141] Dodgson SE Santaguida S Kim S: The pleiotropic deubiquitinase Ubp3 confers aneuploidy tolerance. *Genes Dev.* 2016;30(20):2259–71. 10.1101/gad.287474.116 27807036 PMC5110993

[142] Kwon-Chung KJ Chang YC: Aneuploidy and drug resistance in pathogenic fungi. *PLoS Pathog.* 2012;8(11):e1003022. 10.1371/journal.ppat.1003022 23166494 PMC3499572

[143] Selmecki A Forche A Berman J: Aneuploidy and Isochromosome Formation in Drug-Resistant *Candida albicans*. *Science.* 2006;313(5785):367–70. 10.1126/science.1128242 16857942 PMC1717021

[144] Sionov E Lee H Chang YC: *Cryptococcus neoformans* overcomes stress of azole drugs by formation of disomy in specific multiple chromosomes. *PLoS Pathog.* 2010;6(4):e1000848. 10.1371/journal.ppat.1000848 20368972 PMC2848560

[145] Hope EA Amorosi CJ Miller AW: Experimental Evolution Reveals Favored Adaptive Routes to Cell Aggregation in Yeast. *Genetics.* 2017;206(2):1153–67. 10.1534/genetics.116.198895 28450459 PMC5499169

[146] Lauer S Avecilla G Spealman P: Single-cell copy number variant detection reveals the dynamics and diversity of adaptation. *PLoS Biol.* 2018;16(12):e3000069. 10.1371/journal.pbio.3000069 30562346 PMC6298651

[147] Morard M Macías LG Adam AC: Aneuploidy and Ethanol Tolerance in *Saccharomyces cerevisiae*. *Front Genet.* 2019;10:82. 10.3389/fgene.2019.00082 30809248 PMC6379819

[148] Ma L-J van der Does HC Borkovich KA: Comparative genomics reveals mobile pathogenicity chromosomes in *Fusarium*. *Nature.* 2010;464(7287):367–73. 10.1038/nature08850 20237561 PMC3048781

[149] Goodwin SB Ben M'Barek S Dhillon B: Finished Genome of the Fungal Wheat Pathogen *Mycosphaerella graminicola* Reveals Dispensome Structure, Chromosome Plasticity, and Stealth Pathogenesis. *PLoS Genet.* 2011;7(6):e1002070. 10.1371/journal.pgen.1002070 21695235 PMC3111534

[150] Schotanus K Soyer JL Connolly LR: Histone modifications rather than the novel regional centromeres of *Zymoseptoria tritici* distinguish core and accessory chromosomes. *Epigenetics Chromatin.* 2015;8:41. 10.1186/s13072-015-0033-5 26430472 PMC4589918

[151] Peng Z Oliveira-Garcia E Lin G: Effector gene reshuffling involves dispensable mini-chromosomes in the wheat blast fungus. *PLoS Genet.* 2019;15(9):e1008272. 10.1371/journal.pgen.1008272 31513573 PMC6741851

[152] Möller M Habig M Freitag M: Extraordinary Genome Instability and Widespread Chromosome Rearrangements During Vegetative Growth. *Genetics.* 2018;210(2):517–529. 10.1534/genetics.118.301050 30072376 PMC6216587

[153] Habig M Quade J Stukenbrock EH: Forward Genetics Approach Reveals Host Genotype-Dependent Importance of Accessory Chromosomes in the Fungal Wheat Pathogen *Zymoseptoria tritici*. *mBio.* 2017;8(6):e01919–17. 10.1128/mBio.01919-17 29184021 PMC5705923

[154] Lieberman-Aiden E van Berkum NL Williams L: Comprehensive mapping of long-range interactions reveals folding principles of the human genome. *Science.* 2009;326(5950):289–93. 10.1126/science.1181369 19815776 PMC2858594

[155] Duan Z Andronescu M Schutz K: A three-dimensional model of the yeast genome. *Nature.* 2010;465(7296):363–7. 10.1038/nature08973 20436457 PMC2874121

[156] Burton JN Adey A Patwardhan RP: Chromosome-scale scaffolding of *de novo* genome assemblies based on chromatin interactions. *Nat Biotechnol.* 2013;31(12):1119–25. 10.1038/nbt.2727 24185095 PMC4117202

[157] Smukowski Heil C Burton JN Liachko I: Identification of a novel interspecific hybrid yeast from a metagenomic spontaneously inoculated beer sample using Hi-C. *Yeast.* 2018;35(1):71–84. 10.1002/yea.3280 28892574 PMC5771821

